# Spatial Variation in Foraging Behaviour of a Marine Top Predator (*Phoca vitulina*) Determined by a Large-Scale Satellite Tagging Program

**DOI:** 10.1371/journal.pone.0037216

**Published:** 2012-05-21

**Authors:** Ruth J. Sharples, Simon E. Moss, Toby A. Patterson, Philip S. Hammond

**Affiliations:** 1 Sea Mammal Research Unit, Scottish Oceans Institute, University of St Andrews, St Andrews, Scotland, United Kingdom; 2 The Institute for Marine and Antarctic Studies, University of Tasmania, Hobart, Australia; 3 CSIRO Wealth from Oceans Research Flagship, Hobart, Australia; Texas A&M University-Corpus Christi, United States of America

## Abstract

The harbour seal (*Phoca vitulina*) is a widespread marine predator in Northern Hemisphere waters. British populations have been subject to rapid declines in recent years. Food supply or inter-specific competition may be implicated but basic ecological data are lacking and there are few studies of harbour seal foraging distribution and habits. In this study, satellite tagging conducted at the major seal haul outs around the British Isles showed both that seal movements were highly variable among individuals and that foraging strategy appears to be specialized within particular regions. We investigated whether these apparent differences could be explained by individual level factors: by modelling measures of trip duration and distance travelled as a function of size, sex and body condition. However, these were not found to be good predictors of foraging trip duration or distance, which instead was best predicted by tagging region, time of year and inter-trip duration. Therefore, we propose that local habitat conditions and the constraints they impose are the major determinants of foraging movements. Specifically the distance to profitable feeding grounds from suitable haul-out locations may dictate foraging strategy and behaviour. Accounting for proximity to productive foraging resources is likely to be an important component of understanding population processes. Despite more extensive offshore movements than expected, there was also marked fidelity to the local haul-out region with limited connectivity between study regions. These empirical observations of regional exchange at short time scales demonstrates the value of large scale electronic tagging programs for robust characterization of at-sea foraging behaviour at a wide spatial scale.

## Introduction

Harbour seals have a widespread distribution across the Northern Hemisphere, from temperate to polar regions. There are five recognised sub-species; our study focuses on *Phoca vitulina vitulina,* which occurs in the eastern Atlantic, from Brittany (France) to the Barents Sea (Russia) and as far north as Svalbard (Norway). This paper presents an analysis of movement and foraging data from harbour seals tagged at seven regions around Britain; some of the most extensive data collected on harbour seal movements to date.

Improving our understanding of the movements of harbour seals is of interest ecologically but is also important to conservation and management. Subpopulations of *P. v. vitulina* in northern Britain have recently declined by around 50% in less than 10 years [1]. The cause of this decline is unknown. There have also been large scale epizootics of Phocine Distemper Virus (PDV) killing approximately 20 and 30 thousand harbour seals in 1988 and 2002, respectively, across Europe. Data on movements allow the level of interchange and hence spread of disease to be modelled [2].

Although British harbour seal populations are monitored via aerial surveys of the numbers present at haul-outs [1], little is known of their at-sea movements. Harbour seal movements have been studied in the Moray Firth and Orkney using VHF telemetry [3]–[5]. However, the ‘line of sight’ requirement for this technology, although useful for studying movements in coastal environments, does not allow observations of long range movements or movements to unobserved sites. Satellite telemetry does permit such observations of long range movements and is more appropriate for large scale studies such as presented here.

Obtaining data on movement patterns from a greater spatial domain is important for several reasons. Determining how often individuals move to other subpopulations is critical for developing realistic models that help understand the spread of epizootics [2]. Movement data are also valuable for understanding population dynamics and the persistence of local populations [6].

Knowledge of movements and at-sea usage data are useful in broadening our understanding of the distribution of ecosystem impacts of top predators. Feeding in both pelagic and benthic habitats, harbour seals are considered generalist predators and take a wide variety of fish, cephalopods and crustaceans (e.g. [7], [8]). Their diet is highly varied and seals from different areas show marked differences in prey, although a few species generally dominate the diet in any one area at any one time of the year [9]–[11]. This dietary variation is associated with seasonal changes of prey abundance [12]–[14]. Regional and seasonal variation in diet implies that foraging behaviour and movements to obtain prey may also be expected to vary regionally and seasonally.

Sex and size of harbour seals have been found to be predictors of foraging trip distance and duration, in the Moray Firth (Scotland) in summer [15]. Conversely, harbour seals in inshore waters of western Scotland displayed no sexual difference in foraging duration, although females were found to be foraging further [16]. Studies of other pinniped species have also shown degrees of sexual segregation in foraging behaviour and areas, however these species exhibit greater sexual dimorphism than harbour seals [17], [18]. Previous harbour seal studies have not collected movement data from such a large representative selection of regional populations and have thus been unable to characterize the true variability in foraging strategies. Lacking this essential understanding of how variable foraging strategies are between regions, genders or individuals could obscure the drivers of population change and ultimately hinder conservation and management decision making.

We expect seals to forage in a manner optimised for local conditions. In this study we concentrate on characterizing variability in foraging duration and trip length, which may be related to a combination of factors. These might include intrinsic factors such as a seal size, age, sex or body condition. Alternatively, local habitat characteristics such as substrate or water-depth might shape foraging behaviour. Interactions between habitat and intrinsic physiological factors could also influence the foraging strategy employed by a seal (e.g. only at a certain size or condition may foraging be feasible in deeper water see [22]). On the other hand, prey abundance, which is likely to vary seasonally, may be a better predictor of foraging strategy. The relative influence of these factors is likely to vary through time and space and this makes understanding the drivers of foraging strategy challenging. The collection and analysis of widespread data on movements and thus foraging is a prerequisite for understanding how top predators interact with their environment.

In this paper, we present results from a tracking study of 118 harbour seals from seven major populations around Britain. We describe regional foraging movements and between region movements. We investigate regional and seasonal foraging behaviour and seek to identify whether intrinsic variables such as sex, length and a proxy for body condition are good predictors of movement characteristics in harbour seals or whether the region and therefore habitat where the seals were captured and forage plays a greater role.

## Methods

### 2.1 Ethics Statement

Seal capture and handling was conducted under the terms of licences 60/3303 (Foraging, physiology and abundance of seals) and 60/4009 (Ecology, physiology and population dynamics of UK seals) issued by the UK Home Office under the Animals (Scientific Procedures) Act 1986.

Seal capture and handling was also authorised by the UK Scottish Office under annual licences given to the Sea Mammal Research Unit in accordance with the UK Conservation of Seals Act 1970.

### 2.2 Study Area Selection

**Figure 1:**
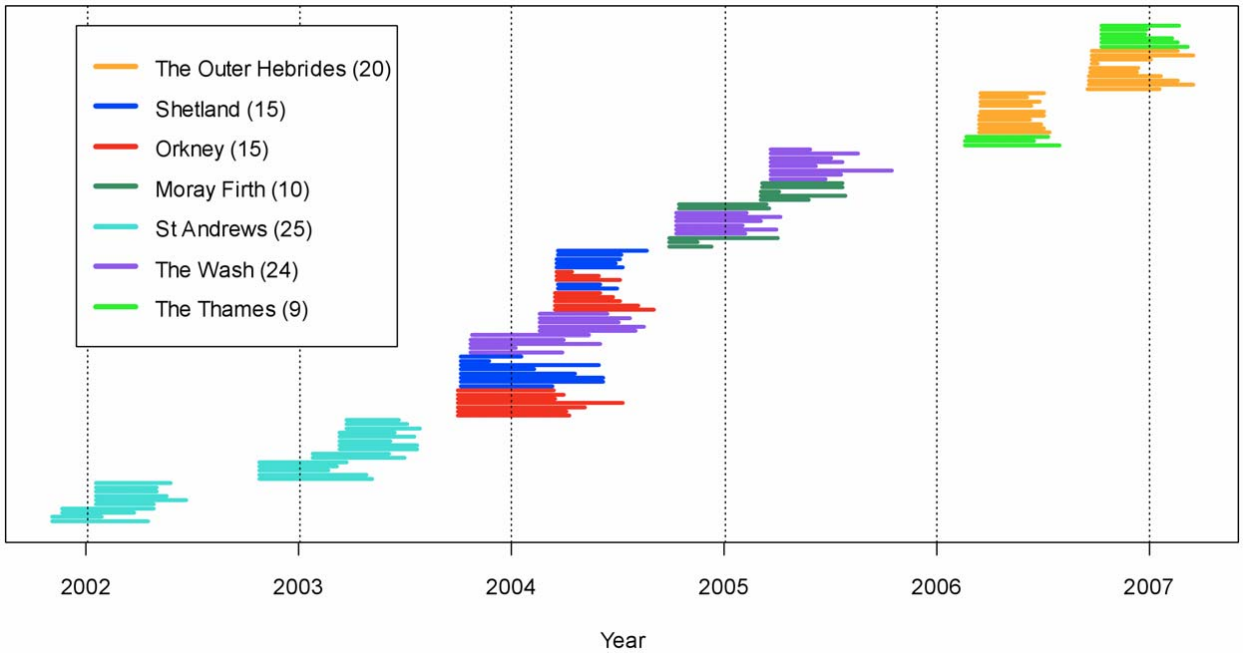
Tag deployment distribution and longevity. Individual tag deployments by site. The length of each line shows the duration of deployment for an individual seal.

Harbour seals were studied in seven regions around Britain. These areas represent the major British harbour seal population [1], which we refer to by their tagging location (see Figure 1 and Table 1). The regions were: (i) the Moray Firth and (ii) St Andrews Bay in eastern Scotland; (iii) the Orkney and (iv) Shetland islands north of mainland Scotland; (v) the western isles of the Outer Hebrides; and (vi) The Wash and (vii) the Thames estuary, both on the eastern English coast (full details of the releases are given in Appendix S1).

These sites span a wide range of at-sea foraging and haul-out habitats. Like many pinnipeds, harbour seals periodically haul out on land to pup and moult, and between foraging bouts. Haul-out habitats in the islands to the west and north of Scotland typically consist of rocky intertidal areas, whereas intertidal sandbanks are used on much of the east coast of Scotland and England. Foraging habitat also varies spatially. Relatively deep water, characterized by a high proportion of rocky substrate, occurs in close proximity to haul-outs in the northern and western regions of the British harbour seal distribution. In contrast, the western North Sea consists of relatively shallow water with low-relief and sedimentary substrates.

**Table 1:** Number and type of tags deployed in different areas throughout the UK and number of trips recorded.

Tagging Region	Number of harbourseals tagged andtype of tag	♂	♀	Average numberof locationsreceived (SD)	Average tagging duration(days)(SD)	Number of trips recorded
Outer Hebrides	20 (18 SRDL, 2 GPS)	8	12	1717 (972)	111 (34)	877
Shetland	15 SRDL	7	8	1596 (1032)	133 (70)	840
Orkney	15 SRDL	7	8	1528 (599)	141 (65)	877
Moray Firth	10 SRDL	6	4	1106 (461)	110 (50)	142
St Andrews Bay	25 SRDL	13	12	1272 (276)	125 (25)	559
The Wash	24 SRDL	11	13	859 (319)	132 (39)	440
The Thames	9 (8 SRDL, 1 GPS)	9	0	1168 (342)	123 (30)	649
					**Total**	**4384**

### 2.3 Catching, Handling and Tagging of Seals

A range of techniques were used to catch seals depending on local habitat conditions. Where seals hauled out on intertidal sandbanks (Moray Firth, St Andrews Bay, The Wash and the Thames), most seals were caught using a long net (120 m), set from a boat around the haul-out site. The net formed a barrier within which seals were trapped as they fled to the water [20]. The net was hauled to shore and animals were transferred to smaller hoop nets until anaesthetized (see below). When there was a strong prevailing current, when many grey seals were in proximity to the harbour seal haul-out prohibiting net setting, or when there were too few animals to make net deployment worthwhile, the ‘rush and grab’ technique was used as detailed below.

Seals hauled out in rocky habitats (e.g. Outer Hebrides, Shetland and Orkney) were mostly caught using the ‘rush and grab’ technique in which a small inflatable vessel was driven quickly up to the haul-out area, deploying people carrying hoop nets to catch animals before they reached the water. If ‘rush and grab’ was not successful tangle nets were deployed in the area around the haul-out sites. This tended to be in areas where seals can reach deep water before capturers can get close enough in a boat, or else shallow rocks prohibited running a vessel at speed to the haul-out site. These loose-mesh nets were deployed at different angles around the haul-out sites to entangle seals when they swam into them. Buoys attached to either end of the net indicated when an animal was captured. Captured seals were untangled and transferred into the boat and restrained with a hoop net.

All seals were weighed and then anaesthetized using 0.05 ml of Zoletil per 10 kg body mass delivered intravenously [21]. Measurements of girth and standard length (straight distance from the nose to the tail) were taken for each seal and sex recorded. The ratio of mass to length was used as an approximate measure of body condition or energy reserves as for grey seal weaned pups by [22].

Transmitters were attached to the fur on the neck of the seal at the base of the skull using a fast setting two-part epoxy adhesive [23]. Seal capture and handling was conducted under the terms of licences issued by the UK Home Office under the Animals (Scientific Procedures) Act 1986 and the Scottish Executive under the Conservation of Seals Act 1970.

### 2.4 Satellite Transmitter Protocols

Satellite-Relay Data Loggers (SRDLs) comprise a data logger interfaced to an Argos transmitter unit and a pressure (depth) and conductivity (submergence) sensor (see http://www.smru.st-andrews.ac.uk/protected/downloads/SRDL9000X.pdf). The SRDLs measured 100×70×45 mm (excluding the 150 mm antenna) and weighed 305g. The SRDLs collected, compressed and transmitted data via the Argos system (System Argos, Toulouse, France); a detailed description is given in [24]. Data were transmitted on location, diving depth, swimming speed, and proportion of time hauled out on land [24], [25].

Data recorded by the SRDLs provided information on the time a seal spent at the surface, diving or hauled-out throughout any given 2 h time period. These activity categories were assigned according to the following rules: if the tag was dry (i.e. not submerged) for more than 10 minutes the seal was recorded as hauled-out; a haul-out period was determined to have ended when the tag became wet or submerged for a minimum of 16 s and at a depth greater than 2 m. Otherwise, a seal was classified as ‘diving’ at depths greater than 2 m and ‘at the surface’ at depths less than 2 m. The average and maximum dive depth within a 2 h summary period were also transmitted. Data were stored and transmitted at random to prevent bias in the data received.

GPS phone tags were deployed on three seals in the Outer Hebrides and the Thames (see http://www.smru.st-andrews.ac.uk/protected/downloads/GPS_Phone_Tag22.pdf). These tags were programmed to send all information detailed above for SRDLs, but used Fastloc GPS technology which enables higher quality positions to be obtained using GPS satellites over very short surface intervals [26]. These tags are able to transmit greater amounts of higher resolution data by utilising the mobile phone GSM network, which provides far greater transmission bandwidth than service Argos.

### 2.5 Movement Analysis

#### 2.5.1 - Filtering of locations and track construction

Argos locations are subject to varying degrees of spatial error. Service Argos reports that 68^th^ percentile accuracies range from 150 to >1000 m (Service Argos 1996), however real data collected from seals fitted with both Fastloc GPS and ARGOS tags show that it is common for errors to be an order of magnitude greater than this [27]. With such large degrees of error associated with the majority of locations it was necessary to filter the data to enable meaningful interpretation of movement patterns. We therefore filtered the location data using a hybrid Speed/Kalman filter [28]. Locations obtained by this method are hereafter simply referred to as a Kalman filtered locations. This method first applies a speed filter [24], which removes implausible locations by setting a speed threshold for plausible speed of travel and applying an iterative forwards/backwards average procedure. In this instance the threshold speed applied was 5.5 km.h^−1^. The remaining data are assumed to contain errors approximately distributed as a two dimensional normal distribution, allowing the Kalman filter to be applied (see [28]). Because the SRDLs transmit opportunistically when the seals are at the surface or hauled-out, the number of locations received per day varies and transmissions arrive irregularly. Therefore, Kalman smoothing [28], was used to interpolate a track with regular 2 hourly locations. From Argos data collected from grey seals (*Halichoerus grypus*), it was found that the combined speed/Kalman filter yielded root-mean square errors between 6–12 km calibrated against concurrently collected GPS data [31]. Therefore, the level of error in the filtered and smoothed tracks was typically much smaller than the large-scale movements of harbour seals.

For GPS phone tags, calibration studies have shown that 95% of locations are accurate to +/−55 m and, even when information from fewer than four GPS satellites is received, the mean error is less than 150 meters [29]. GPS positions were thus far more accurate than Argos locations and were treated as error-free, known locations. The Kalman filter was therefore applied assuming no error to provide 2 hourly interpolated locations thus allowing GPS data to be treated in the same way as data collected from the SRDLs in subsequent analysis.

#### 2.5.2 - Categorising tracking data into individual trips

Because *P. vitulina* is a central place forager, the duration of trips is likely to be a key descriptor of differences in foraging behaviour among regions. Therefore tracks from individuals were classified into a series of individual ‘trips’. This task is not straightforward because of the inaccuracies in location data and also because of the summarized nature of the SRDL diving data (see above and [25]). In this paper ‘trip splitting’ was carried out using the following approach. We assumed that intensively foraging seals will dive to depths in excess of 10 meters within any 2 hourly period. On the other hand, when seals are near to shore in the vicinity of haul-outs, diving will be minimal and largely shallower than the 10 m threshold depth. Accordingly a trip was taken to start when three consecutive 2 hourly summary records having a maximum dive depth of 10 m or more occurred in the dive-summary time series. In this instance, the time of the first 10 m maximum dive depth was taken as the start of the trip. A trip ended when the wet/dry sensor reported the animal as hauled-out (dry for 2 mins). The last trip recorded for each seal prior to the tag ceasing to work was often incomplete if this occurred at sea. Partial trips were therefore removed from the data used for analysis.

We chose to use a depth of 10 m in our trigger to initiate a trip to avoid generating many short trips that would have occurred if a shallower depth had been used. However, this choice is likely to cause some foraging to be missed in shallow water close to haul-out sites (e.g. Lesage et al 1999). Without such additional data on feeding events we cannot quantify how much foraging occurred in shallow waters but we assume here that our choice of a 10 m depth threshold should not impact our conclusions.

In five of the ten tags deployed in the Moray Firth population, haul-out and dive information could not be processed into trips using the approach outlined above because of tag malfunction. Therefore, for these data sets, we used a simple rule that assigned a trip start time when a seal’s estimated location was at least 10 km from a haul-out site and ended when within 10 km of a haul-out site. This was only possible in this area because of the nature of the prolonged distance and duration of these foraging trips (see below). To check this did not cause any significant upwards bias in the overall findings of the study the analysis was repeated on just the five animals tagged in the Moray Firth whose tags were operating correctly and the same patterns were apparent with little difference in the observed mean foraging duration or distance travelled. Note that this simple distance-based rule could not be used to categorise trips for all seals in all areas because for animals that travelled small distances from the haul-out site the errors remaining in filtered tracks were of the same order as the distance travelled.

Other studies have used a distance from haul-out site threshold to trigger the initiation of trips [30] however in this study the level of Argos error prohibits this in areas where seals were travelling very short distances from the haul-out to forage.

Only foraging trips returning to the same region were included in the analysis, because it is not clear to which region a between-region trip should be assigned and because there were only a few between-region movements, as discussed in the Results.

The main focus of this study was to examine differences in the distribution of trip distance and duration when seals were foraging, while accounting for differences in body condition within regions and by time of year. However because movements of seals will be confounded with breeding status, data from breeding season months (June to August) were excluded from analysis allowing us to focus on foraging behaviour alone. During breeding periods, females restrict their foraging range during pupping and lactation [31], while males restrict their foraging range in the mating season which follows [32]. In addition, tags often detached from animals during the breeding season and so a representative sample of males and females was unavailable for all areas for this period. Seals were not tagged in September because transmitters could not be attached during the moult. Therefore analyses were restricted to data from October to May only.

#### 2.5.3 - Statistical Analysis of foraging trip summaries

We investigated whether two indices of foraging effort; the time spent at sea (trip duration) and the distance covered on a foraging trip (trip distance), could be predicted by a range of explanatory variables: the time of year (day of the year calculated as days pre or post 1 October); inter-trip duration (the time between the start of current trip and the end of the previous trip); tagging region; sex; and seal length (a proxy for age). Note that inter-trip duration is not the same as haul-out duration because, by our trip-splitting rule, the seal could remain in shallow (<10 m) water for long periods.

For pinnipeds foraging outside the breeding season, it may not be immediately clear whether the duration of a foraging trip or the distance covered per trip is a better indicator of foraging effort, and each has strengths and weaknesses. Trip distance has the advantage of being spatially explicit but, unlike trip duration, its accuracy depends on the accuracy of Argos correction routines. In contrast, the accuracy of trip-duration depends on the veracity of the criterion for when a trip starts and stops, although diving behaviour was more reliably measured than location – particularly at small scales. Aside from these observational aspects, distance covered may under-represent foraging effort if the foraging strategy entails short distance trips because animals could forage intensively for long periods close to the haul-out site yielding relatively small measures of trip distance. On this basis, trip duration may be a better gauge of changes in foraging effort. Therefore, we use trip duration as an index of foraging effort but also report the regional differences in trip distances.

For each trip (as defined above) we calculated *T*_dur_, the foraging trip duration calculated as the elapsed time between the start and end of a foraging trip. We also calculated *T*_dist_, the foraging trip distance calculated as distance between position locations. For the reasons outlined above and the non-independence of *T_dur_* and *T_dist_* (see Results) only *T_dur_* was analysed using statistical models.

The strictly positive distribution of *T*_dur_ mandates the use of statistical models capable of handling non-Gaussian response variables and also capable of modelling individual variability between seals. We also needed to allow for the possibility of auto-correlation in *T*_dur_. Therefore we used Generalized Additive Mixed Models (GAMMs). Our most complex or ‘saturated’ model can be expressed as
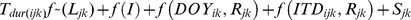
(1)


and included seal length (*L*), sex (*S*), inter-trip duration (ITD), day-of-the-year (DOY) and region (*R*) as explanatory variables of trip duration. The term DOY was calculated as days after 1 October, which indicated the start of seasonal data collection after breeding and moult period data had been discarded. In model (1) the subscript *i* indexes individual foraging trips, *j* indexes seals and *k* indexes the seven different tagging regions. In model (1), terms with two covariates denote interactions. The notation *f*(.) denotes non-parametric smooth terms in the GAMMs which were modelled using thin-plate splines [39]. We also included a random effect on individuals within region to accommodate regional variation among individuals. Without a random effect on individuals within region, one would need to estimate a parameter for each individual seal which would preclude conclusions being drawn on the general behaviour of individuals by over-fitting the data.

Day of the year was included as an integer which equalled zero on 1 October, was modelled as a non-parametric smooth term and was assumed to interact with region, *R*. This means that each regional subset of the data was allowed to have its own temporal progression of trip-durations, but these were assumed to be smoothly varying (unknown) functions. Similarly the effect of inter-trip duration was allowed to vary between regions. Finally the possibility of autocorrelation in trip-duration was modelled by a first-order autoregressive error structure, hereafter referred to as AR(1).

Because GAMMs were fitted using Penalized Quasi-Likelihood estimation [33], commonly used model selection criterion, such as AIC are also approximations. Following [34] we used Quasi-Akaike’s Information Criterion (AIC) to guide model selection and considered simpler variants of (1). Our model selection strategy was, therefore, first to fit the most complex model and successively to remove terms or autocorrelation structure and examine the effect on the significance of each term and the overall model QAIC (see Table 2). Note this applied both to inclusion of potential explanatory variables and autocorrelation structures. Within a given candidate model the inclusion of fixed effects was guided by Wald tests on fixed effects [34]. As a further check, the importance of particular covariates was also qualitatively assessed by examining model predictions both including and excluding particular covariates. Another way that the role of the intrinsic variables (sex, length and inter-trip duration) was examined was to fit separate models for trip duration for each region. This is a statistically cumbersome approach but in this instance confirmed the results of the models fitted to the data from all regions simultaneously (see below).

**Table 2:** Specification of GAMMs of trip duration T_dur_ and model selection process.

Model Code	Model Structure	AR(1)	df	QAIC	ΔQAIC	Adjusted R^2^
M1	T_dur_ ∼ *f*(ITD) + *f*(DOY | Region)+*f*(Length | Sex)	Y	31	11887.5	11.35	0.23
M2	T_dur_ ∼ ITD + *f*(DOY| Region)	N	24	11898.46	22.215	0.22
M3	T_dur_ ∼ month + Region + *f*(Length | Sex)	N	20	12281.59	405.34	0.17
M4	T_dur_ ∼ *f*(ITD) + *f*(DOY| Region)	Y	26	11876.25	0	0.23
M5	T_dur_ ∼ *f*(DOY | Region)	Y	24	12261.81	385.56	0.2
M6	T_dur_∼ *f*(ITD) + *f*(DOY| Region)+ Length | Sex	Y	28	11881.85	5.5	0.23

Here *f*(.) denotes a non-parametric smooth and ‘|’ denotes an interaction between two terms (see equation (1) and text for key to the model terms). All models included a random effect on individual seals. Models where AR(1) autocorrelated errors were incorporated are marked ‘Y’. In the model selection process we followed the following steps: The most complex model M1 (equation 1) was fitted first. In M2, the “biological” variables length and sex were removed, and also the autocorrelation term. Model M3 considered if a complex smooth was necessary for DOY or could instead be replaced by a single Month term. Model M4 considered only non-biological factors but included AR(1). In M5 we further removed inter-trip duration to determine if only DOY interacting with Region were sufficient. Finally in M6 we considered a length effect nested within Sex without smoothing as a final test against M1. Using QAIC and significance tests on individual factors we selected M4 from the candidate set and we found that models that did not include both AR(1) errors or inter-trip duration were very lowly ranked (i.e. much larger QAIC values).

### 2.6 Investigating Regional Differences in Condition

The body condition of an individual seal might determine its ability to remain foraging at sea. We investigated whether there were differences in condition factor (length (cm)/mass (kg)) among different regions. This was important as differences in condition within region may alias any other regional signal. Initial inspection showed approximate linearity in the relationship log(mass) at length. Therefore on the basis of parsimony, we a used linear model
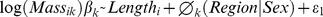
(2)


to determine if there were regional differences in the Length/Mass relationship via the coefficient *β_k_*, and via the interaction of Region/Sex we tested whether the slope of this relationship differed among regions and between sexes.

## Results

### 3.1 Tag Deployments and Data Retrieval

**Figure 2:**
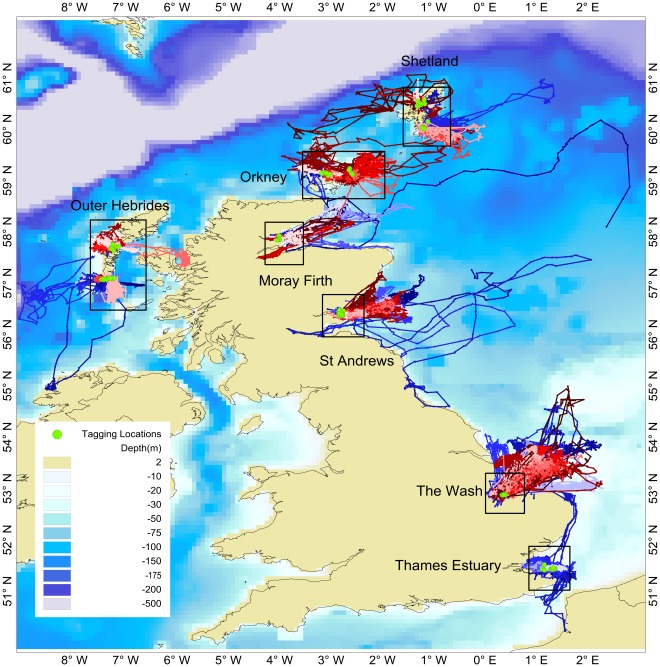
118 smoothed telemetry tracks and capture locations. Smoothed and interpolated tracks of all 118 seals, males in shades of blue, females in shades of red. Green circles show where animals were captured and the major divisions of the data into regions are shown as labelled boxes. ETOPO-2 bathymetry data (ETOPO-2USDC, 2006) in meters are shown.

In total, 118 harbour seals were caught and tagged between November 2001 and October 2006. Of these, 115 seals were tagged with Satellite Relay Data Loggers (SRDLs) and the remaining three were tagged with GPS fastloc tags. The number of tags from each of the seven tagging locations is shown in Table 1 (See Appendix S1 for full tag deployment details). Figure 1 shows the year of study and longevity of deployed tags. In each region (see Figure 2), tags were deployed on approximately equal numbers of males and females, with the exception of the Thames where only males were able to be caught and tagged. A total of 157300 locations (145051 from SRDLs and 12249 from GPS Fastloc tags) were received from seals during the course of the study amounting to seals being tracked for a total of 14991 seal days. The SRDL tags gave a mean of 1272 (Standard deviation, SD: 581) locations per individual and GPS tags gave a mean of 2450 (SD: 1728) locations per individual. The latter provided higher resolution data both in the accuracy of locations and in the number of locations transmitted; however, this was dependent on individual behaviour and availability of GSM mobile phone coverage in the tagging location. Tags transmitted for a mean of 126 days (SD: 66.11, range: 9–285 days). A mean of 10.26 (SD: 3.15) locations were received per day for the SRDL tagged seals and 18.42 (SD: 10.70) per day for GPS Fastloc tagged seals.

### 3.2 General Movement Patterns and Regional Connectivity

**Figure 3:**
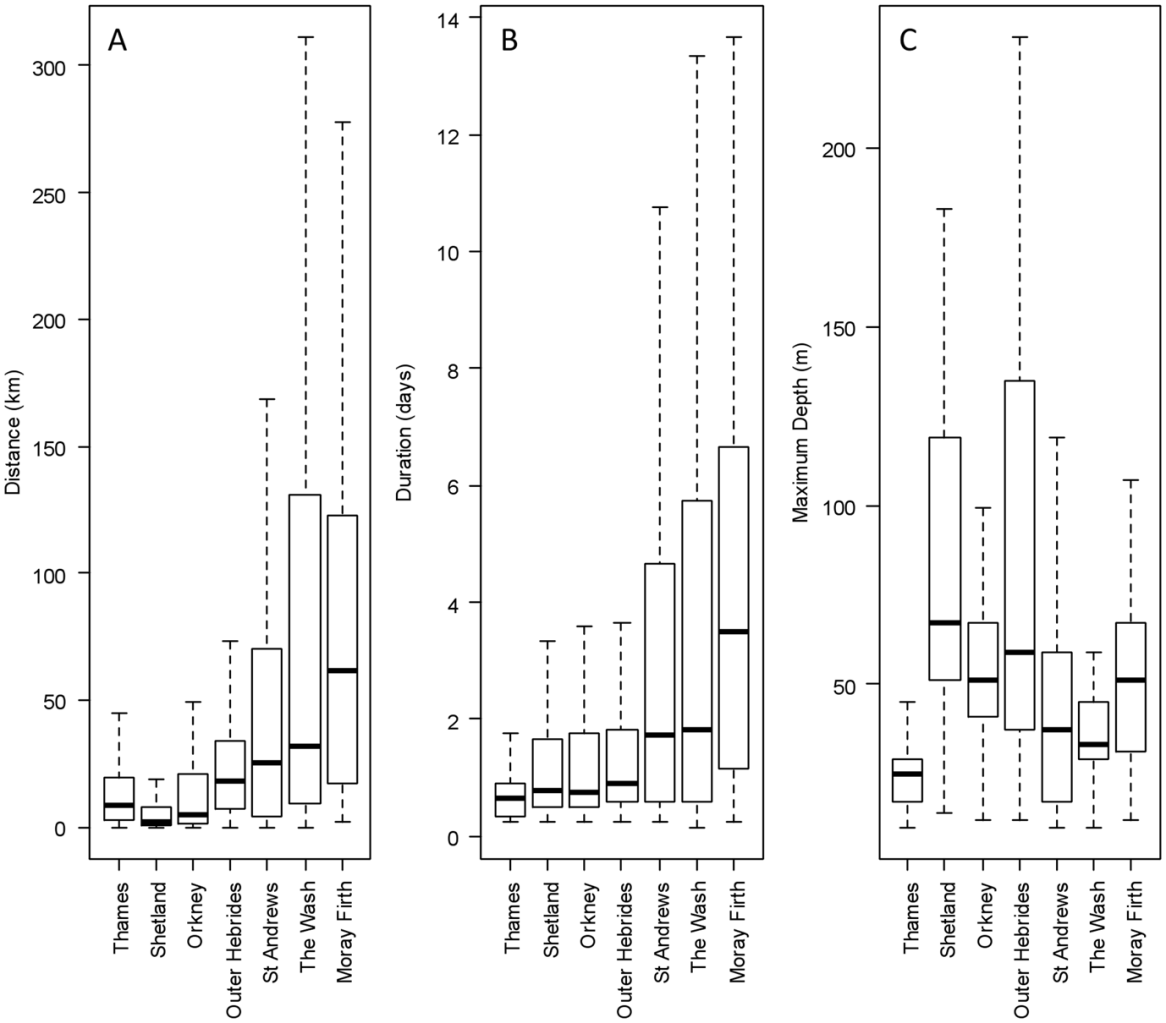
Regional distribution of trip distance, duration and dive depths. Distributions of trip duration, trip-distance and maximum dive depth per trip for each region. These plots highlight the regional differences in foraging.

Harbour seal movements were not restricted to near-shore waters; seals often undertook lengthy trips to offshore locations (Figure 2). Regional differences were apparent in the distances travelled from the haul-out sites to likely foraging areas. Seals on the east coast of the UK (Moray Firth, St Andrews Bay and The Wash) made some of the most wide-ranging trips, whereas animals from the Northern Isles (Orkney and Shetland) and Outer Hebrides generally made shorter distance trips (Figure 3). There was, however, a large degree of individual variation in movement that obscured the pattern of regional differences (Figure 3). On average, seals in the Moray Firth made the longest foraging trips (100.6 km, SD = 129.7 km). However some seals from The Wash made repeated foraging trips of more than 200 km but the large degree of individual variation resulted in a lower average trip distance (mean: 86 km, SD = 111 km). Seals from Shetland, Orkney and the Thames had average foraging trip distances between 11 and 21 km (Figure 3).

The majority of animals were site-faithful in their repeated use of the same or nearby haul-out sites, however a small proportion of animals did travel between regions and even between countries (Figure 2). In the Outer Hebrides, one animal travelled to a haul-out site in Donegal, Northern Ireland more than 450 km away before returning to the area in which it was captured. Another Outer Hebrides animal relocated to haul-out and forage in the Inner Hebrides 140 km away. Of the seals tagged in Orkney, one female moved repeatedly between Orkney and Shetland, a distance of more than 220 km each way, hauling out in both island groups. A male tagged in Orkney also travelled to haul-out sites on the northern coast of mainland Scotland, 75 km to the south. One young male tagged in St Andrews Bay travelled to Leith docks (near Edinburgh) where it remained for 3 weeks, and then travelled south to the docks in Newcastle-upon-Tyne where it remained for several months (reportedly scavenging from fishing boats). An animal tagged in the Thames travelled south across the English Channel to haul-out near Saint-Valery-sur-Somme, France, before moving to haul-out and forage in The Wash.

Also apparent are what appear to be long distance exploratory movements into the North Sea (Figure 2). Movements at this scale could provide some degree of interchange with populations of seals hauling out on eastern North Sea coasts. A male tagged in the Moray Firth made one trip 480 km into the North Sea to within 150 km of Norway before travelling south at which point the tag ceased transmitting, a total distance of 850 km. Two males tagged in St Andrews Bay made similar but shorter trips east into the North Sea.

Patterns of regional trip durations were replicated in the distributions of trip distance, with long duration trips being made on the east coast of the UK and short trip durations in the Northern Isles and Outer Hebrides (Figure 3, Appendix S2). Moray Firth, The Wash and St Andrews Bay animals tended to make longer distance movements of longer duration. In contrast, animals foraging from the Outer Hebrides, Shetland, Orkney Islands, and from the Thames made much shorter distance and duration trips. The average duration of trips ranged from 4.5 days in the Moray Firth to less than 1 day in the Thames (Figure 3). Strong patterns were also apparent in the regional distributions of maximum depth encountered by seals within a trip (Figure 3); seals from the Outer Hebrides, Shetland and Orkney dived deeper than those in the North Sea and English Channel habitats (Moray Firth, St Andrews, The Wash and the Thames). Deep water foraging habitat is simply unavailable in close proximity to these haul out locations.

**Figure 4:**
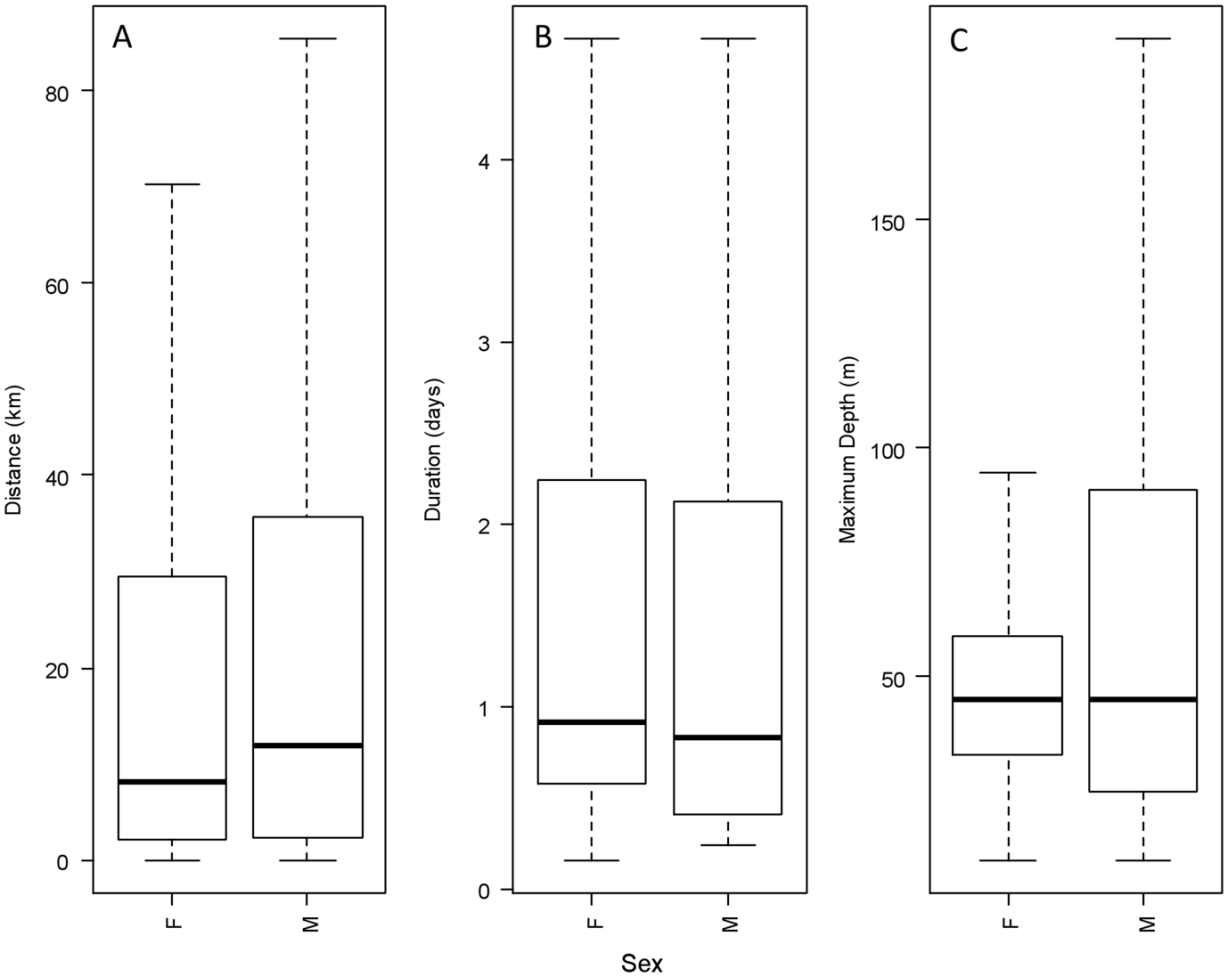
Difference in trip duration, distance and maximum depth by sex. Box-plot representation of distributions of trip-duration, distance-travelled-per-trip and maximum dive depth within a trip between the sexes. Considerable overlap is displayed between the sexes. While in some populations male seals consistently appeared to travel further, sex did not turn out to be a good predictor of trip-duration or distance travelled in general.

Comparison of trips made across regions stratified by sex (Figure 4) shows that males travelled slightly further than females on average (males: mean = 36.6 km, SD = 74.5; females: 30.7 km, SD = 61.2). This was not the case for duration of foraging trips, with male and female foraging trip duration being similar in mean trip duration and also in the spread of the data. Further examination of differences between sexes is considered within the statistical model results below.

**Figure 5:**
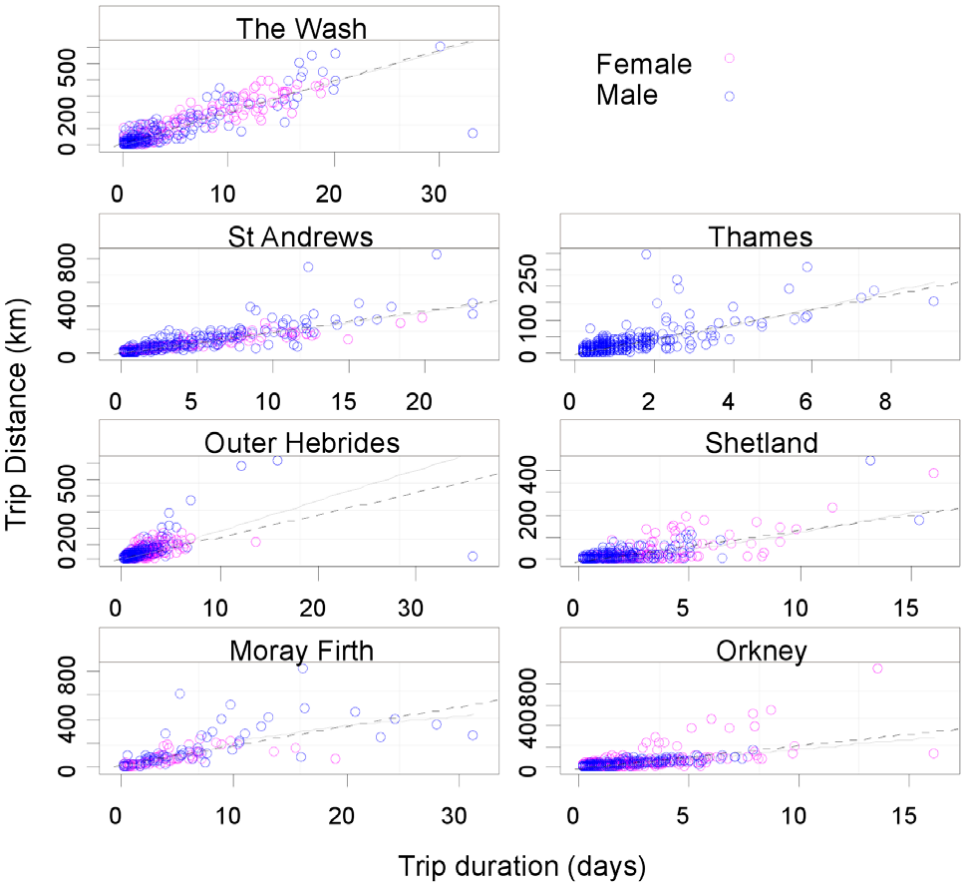
Relationship of trip distance and duration by sex for each region. Plots of distance travelled per trip against trip duration shown for each region. Pink circles show data from female seals and blue circles show males. Lines are LOESS smoothers (span = 1) which were added to highlight trends. Apparent is the large degree of variability within region particularly for relatively long trip durations. Note that the horizontal axis varies among plots, also highlighting the differences in the range of trips among regions.

The relationship between trip distance and duration was approximately linear (Figure 5). However, the strength of this relationship was again regionally dependent and variability in trip distance often increased with larger durations. For example, relatively lengthy periods spent at sea often involved seals remaining close to the haulout locations. The trip distance/duration relationship was least variable for The Wash and St Andrews. For the other sites there were obvious departures from the linear relationship, most conspicuously for Moray Firth seals. Here the male seals tended to cover more distance on foraging trips. However, sex was not a good predictor of foraging trip distance in all regions and turned out to be rejected as an explanatory covariate in the models (see below).

### 3.4 Statistical Analysis of Trip Duration and Distance

**Figure 6:**
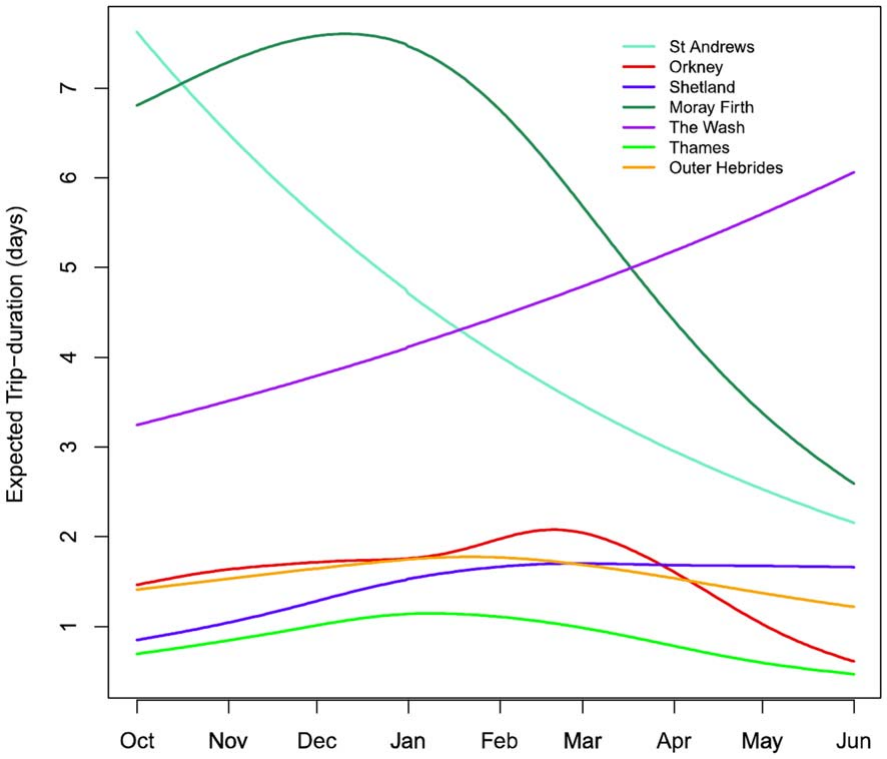
Expected trip durations by region for time of year. Expected trip duration by site through the year as predicted by the selected GAMM (M4 in Table 2). At each site the mean ITD was used for generating model predictions. The plot shows both how longer trips are expected in the Moray Firth, St Andrews and The Wash relative to the other regions and also how the timing of long trips varies through the year.

Comparison of a range of GAMMs resulted in selection of a model containing only inter-trip duration, DOY and the categorical variable region (Table 2). The fitted GAMMs explained a modest amount of the variability in the data (adjusted R^2^ = 0.17–0.23, Table 2) and model diagnostics (not shown) reflected this. Therefore we cannot claim to have identified the key variables explaining foraging trip behaviour. Nonetheless the models were able to draw out distinct and consistent differences in foraging behaviour among regions and temporal changes in foraging in terms of trip duration (Figure 6). Hypothesis tests from the fitted GAMMs indicated that sex but not length was significant (Table 2). However, the QAIC indicated that models were not substantially improved by inclusion of sex or length but instead favoured more parsimonious models and there were only negligible differences in predicted trip durations between sexes. This indicates that differences between the sexes within each site were small compared to the overall differences among regions. Furthermore, differences between the sexes were small even when compared to individual variability within a region. The Moray Firth and St Andrews Bay were the only regions where trip duration appeared noticeably different between the sexes and only between October and November. This pattern was also apparent in the distance travelled per trip.

It is important to note that making valid statistical inference is difficult with non-Gaussian random effects models [35]. As penalized quasi likelihood methods provide only approximate likelihoods, QAIC calculations and hypothesis tests are to be regarded with a healthy degree of scepticism. Generally we found that most reasonably specified candidate models (i.e. those with region, sex, and day of year included) gave similar predictions and these were in accordance with the major patterns of regional difference displayed by the data.

The selected GAMM predicted that irrespective of the time of year, longer trips were expected in The Wash, St Andrews Bay and the Moray Firth relative to other regions. Notable differences were also apparent in the seasonal changes in expected trip duration. The longest trips for Orkney, Shetland and the Outer-Hebrides were predicted for January and February; trip length for the Moray Firth peaked in December. The longest trips in the Thames were predicted for January, although the variation here was smallest of all the regions and trip length could essentially be regarded as constant (Figure 6). Trip durations in St Andrews Bay and The Wash were predicted to increase and decrease smoothly, respectively, with no peaks throughout the non-breeding seasons. While these trends were generally apparent in the data, these predictions may be a result of the smaller sample sizes at either end of the season for these regions.

Inter-trip duration was retained in the selected model of trip duration. Despite the selection of the model with AR(1) correlations between trips, there was little evidence of temporal correlation in either the raw trip duration data or the residuals from the fitted models (AR(1) correlation parameter  = 0.03) which indicates only a small statistical dependence from one trip to the next. However, because inter-trip duration was retained as a significant explanatory variable this may indicate temporal dependence in overall foraging strategies. Additionally, retention of the smooth functions for day of the year suggest intra-annual trends in trip duration. However, there was considerable variation among regions in the timing of peaks in trip duration. The sites in the North Sea (St Andrews Bay, The Wash and the Thames) showed a much larger signal of seasonal variation compared to the Outer Hebrides, Orkney and Shetland. This may indicate that the food resources in these latter regions tend to be more stable.

Estimation of model (2) indicated a strong relationship between weight and length (F = 110.964, p<<0.001, Multiple R^2^ = 0.53), as expected, but also that there were no differences in weight/length relationships between sex (F = 0.28, p = 0.59) or within region (F = 0.67, p = 0.76). Although the sample size (N = 118) is too small to characterize properly the weight-length relationship, it does suggest that within this dataset, condition did not vary significantly among regions and we therefore conclude that the apparent regional differences in foraging strategy were not aliasing regional differences in the condition of the seals. However, this does not preclude other aspects of foraging (such as diving ability or prey selection) being related to individual condition. Nonetheless, these data support the idea that a large component of the variability in foraging behaviour is due to region-specific variables. Additionally, models which did not contain region as a categorical factor variable were ranked poorly relative to models which did. This pattern, in which time of year and inter-trip duration were the most effective explanatory variables was also found when regional subsets of the data were modelled separately. For brevity these results are not given.

## Discussion

The satellite telemetry data presented here provide the most comprehensive picture to date of the foraging distribution and behaviour of harbour seal populations around Britain, the full foraging range and variation were previously undescribed. Few studies have been able to compare movement patterns and foraging behaviour among multiple colonies/regions of a single species [36]. Most obviously illustrated here is that harbour seals around Britain frequently made wide-ranging movements to sea and also transited between regions. Hence, the British harbour seal should not be considered purely as a coastally distributed marine mammal or as consisting of a set of discrete populations. Importantly, our analysis found that extrinsic factors such as region and time of year were better predictors of foraging behaviour than individual intrinsic factors such as size, sex or body condition. From these results we hypothesize that among-region variation in trip duration and distance is dominated by environmental and habitat constraints at a particular site.

### 4.1 Regional Differences

A clear result from this study is that strong regional differences in foraging behaviour exist among harbour seals hauling out around Britain. Animals from the Outer Hebrides, Shetland, Orkney and the Thames made, on average, short distance, short duration foraging trips. Animals hauling out in the Moray Firth, St Andrews Bay and The Wash made much longer distance and duration foraging trips. An earlier study in the Moray Firth (early 1990s) [37] using VHF telemetry found foraging range to be shorter than observed here (15–25 km). However, the differing telemetry techniques prevent us from drawing the conclusion that harbour seals have undergone range-expansion over the last decade. Harbour seals tagged in north west Scotland at two different sites to this study over a similar time period, found 50% of trips were within 25 km of the haul-out sites but that some seals travelled more than 100 km [16].

Studies from other parts of this species’ range found similar variation. For example harbour seals tagged in Prince William Sound, Alaska were found to be moving between 5–25 km from haul-outs to at-sea foraging locations although some sub-adults made wider ranging movements of 300 to 500 km outside the sound where they were tagged [38]. In the St Lawrence River estuary, Canada, animals were found to be foraging close to the shore (<6.1–11.0 km) in shallow water areas during ice free periods - although many made seasonal migrations of 266 km (range 65–520 km) to over-wintering sites [39]. It should be highlighted, however, that distances to foraging areas are presented using different metrics in different studies making direct comparisons difficult. The winter movements of harbour seals in two bays in France have also been compared, and although the data have not been split into foraging trip metrics, 95% of at sea movements were within 5 km at one bay and 50% in the other, with one long range movement of 380 km also recorded [40].

### 4.2 The Relative Effect of Extrinsic and Intrinsic Factors

The intrinsic factors of sex and length explained little of the large variability within regions. This result that the largest differences were mostly among regions is consistent with harbour seals adopting foraging strategies tailored to their local habitat. Obvious differences in habitat exist for animals hauling out on rocky shores in close proximity to deep water (around the north and west of Britain) and those sites where animals mostly haul out on intertidal sandbanks bordering the shallow and gently sloping sedimentary western North Sea (on the east coast of Britain). Relative to shallow water sedimentary sites, animals at the sites bordering deeper water, rocky bottomed sites, tended to undertake foraging trips of shorter distance and duration.

The characteristics of the local habitat as prey refugia may partially explain increased foraging distances in areas of sedimentary habitat. In soft bottom habitats prey are more accessible to predation and thus may be more depleted. Areas with rocky bottom substrates may provide prey refugia preventing as much prey depletion close to the haul-out site.

If we assume that seals aim to minimize energy expended during foraging trips, our results imply that harbour seals hauling out adjacent to shallow soft-sediment habitats need to travel greater distances to find sufficiently productive foraging areas from which they can maintain comparable levels of body condition, and presumably fitness.

Further fine-scale analysis of foraging, such as examination of the diving behaviour of seals, also collected by tags in this study, may elucidate further on the characteristics of preferred foraging areas and whether foraging is largely benthic or pelagic within the study regions.

Studies of foraging in breeding seabirds, another central place forager, have shown an increase in prey size, as well as a reduction in benthic foraging and increase in pelagic foraging, with distance from the colony [41]. In this study of harbour seals, water depth may limit the efficiency of foraging thus increasing the profitability of foraging closer to haul-out sites where water depth is shallower.

The exception to this pattern was the Thames, where, despite being a gently sloping sedimentary site, animals were found to be travelling only short distances to forage. However, only small numbers of harbour seals haul out at this site, likely because it is a relatively poor quality haul-out site; the sandbanks are adversely affected by the prevailing weather and are exposed for only a small proportion of the tidal cycle. The sandbanks in the Thames may represent marginal haul-out habitat for harbour seals and it is possible that limited intra-specific competition for available prey allows the persistence of relatively small populations.

Our measure of body condition did not differ significantly by region suggests that the different foraging strategies employed in different regions achieve similar levels of individual body condition. An important caveat is that our study animals are not representative of the whole population because animals smaller than 50 kg were not tagged and thus juveniles were not represented. Given the observed population declines around Britain [1], this component of the population should be a high priority for future studies. If prey resources are limited, inexperienced juveniles may suffer higher mortality rates because of lower foraging success and may thus not be successfully recruited into the adult population in sufficient numbers to maintain population size.

Highly sexually dimorphic species of pinnipeds such as grey and elephant seals have been shown to exhibit differential movement and space use according to sex [18], [42]. These segregations are thought to be a mechanism to reduce intra-specific competition. While harbour seals are sexually dimorphic, the differences are small compared to those found for grey and elephant seals and we found that sex was a poor predictor of both trip duration and distance.

Differences between the sexes in harbour seal foraging distance and duration have been observed previously in the Moray Firth; however, that study was focused on late spring and early summer months when differences are likely to be most pronounced prior to the breeding season [15]. Pregnancy in females may restrict trip range and duration due to increased travel costs and also increase requirements for rest. In northwest and southwest Scotland sex was found not to be a good predictor of foraging distance, although females were found to be foraging slightly further from the haul-out sites than males [16]. Additionally, spatial separation may also extend to the vertical dimension as larger animals are able to dive deeper for longer [19]. Higher prey abundance at depth might lead to larger males travelling to deeper, and therefore generally more distant, habitats. However, if sufficient prey were available at depths within the maximum dive threshold of all animals, or the travel costs for accessing deeper water locations were too high, these factors may constrain foraging trips to areas closer to haul-out sites. Because sex, size and body condition explained little variation in trip duration, it seems likely that habitat and prey availability in each region are more important determinants of foraging behaviour individual-based limiting factors such as dive capability.

### 4.3 Seasonal Differences and Prey Availability

Time of year was found to be an important variable in explaining variation in foraging trip duration and distance travelled, a result that corroborates previous studies which found that harbour seals around the coast of Britain spend more time away from haul-out sites during the winter months [19], [38], [41]. This seasonal pattern is apparent throughout their range (e.g. [43], [44]) and is presumably driven by changes in foraging behaviour. Indeed, harbour seals tagged in northwest Scotland increased their range in winter months [16]. This also tallies with studies of seasonal variation in the diet of harbour seals around Britain [10], [11], [13] which suggest that animals adapt their foraging behaviour in response to changes in prey availability. The biomass and distribution of prey at a regional scale are largely unknown; better knowledge of prey abundance and how this varies regionally and seasonally would greatly improve our understanding of harbour seal foraging.

The North Sea ecosystem has shifted markedly in recent decades, greatly affecting relative abundances of fish stocks [45]. Catastrophic failures in the breeding success of sea birds have occurred [46] and these large-scale changes are also likely to have affected the foraging behaviour of other marine predators, including harbour seals. The available data are inadequate for an assessment of this but analyses of the data presented here in combination with ongoing telemetry and diet studies at the Sea Mammal Research Unit and fisheries assessments should improve our understanding of how harbour seal foraging behaviour is changing as their prey base changes.

### 4.3 Intra-specific Competition

For central-place foragers, intra-specific competition should play a part in determining the distance animals are travelling to forage as resources closest to the central place are expected to be most depleted. Consequently, in areas where relatively large numbers of harbour seals haul out, animals may be expected to travel further in order to obtain sufficient food. Although there is some suggestion of this in our data, (i.e. animals from the small Thames population did not travel far on foraging trips), there are a number of complicating factors that probably obscure any relationship of increasing distance travelled with increasing population size. Most importantly, we do not have data on prey availability in the different regions. Also each region differs in the area available in which to search for nearby prey; colonies on islands have a larger area of water surrounding the haul-out; colonies within an enclosed coastline have a reduced area of water close to the haul-out. Finally, there are large differences between colonies in the depth of water surrounding the colony, obviously leading to a completely different species makeup in the prey resources.

Intra-specific competition can also result in sex and age specific foraging strategies [46], [47] although, as discussed above (4.2), sex explained little of the variation in trip distance and duration observed here and a limited range of ages were tagged in this study.

### 4.4 Inter-specific Competition

Regional differences in foraging behaviour could be influenced by competitive interactions with other species and competition with grey seals has been suggested as contributing to recent harbour seal declines. Grey seals have been tagged throughout their range around Britain [47]–[49] and comparisons between at-sea movements of the two species should help to give a better understanding of the spatial overlap in foraging. In St Andrews Bay and at the Farne Islands (northeast England), 88% of grey seal trips returned to the same haul-out site and return trips had a mean maximum extent of 39.8 km from the haul-out site [50], thus indicating that foraging is concentrated in the coastal zone thereby showing the potential for considerable overlap with harbour seal space use.

A study comparing foraging of harbour and grey seals in the inner Moray Firth did find dietary and spatial overlap in the foraging of the two species although grey seals utilized a wider area and generally travelled further [5]. A similar range and similar seasonal variation in prey species were observed in the diet of harbour seals in The Wash (1990–92) and of grey seals at a site 75 km to the south (1985), although the dominant species in the diet differed [13], [47]. The at-sea space use of grey seals around Britain has previously been modelled [48]; updating this and applying comparable techniques to this harbour seal data-set will allow the degree of spatial overlap between the two species to be described throughout Britain and should improve our understanding of the potential for competition for prey.

### 4.5 Predation

Unlike in other parts of their range, natural predators of seals are rare in British waters; however observations of predation on harbour seals by killer whales have been observed in northern and western Scotland, primarily in the pupping season and most frequently in northeast Scotland and Shetland [51]. However, predation events in the open North Sea adjacent to haul-out sites along the east coast of Britain (Moray Firth, St Andrews Bay and The Wash) are much less likely to be observed. The extent of overall killer whale predation pressure and whether or not it has changed is unquantifiable from data currently available but it is conceivable that it could influence harbour seal foraging behaviour. Large-scale ecosystems shifts such as documented for the North Sea [49] have affected killer whale foraging elsewhere. The collapse of sea otter, seal and sea lion populations in southwest Alaska have been linked to changes in killer whale predation driven by changes in the abundance of other prey species [52]. While it is not possible to draw such inferences around Britain, and the northern North Sea in particular, it is conceivable that killer whale predation pressure has an influence on harbour seal foraging behaviour and abundance.

### 4.6 Concluding Remarks

Our study has shown that harbour seals forage more extensively in offshore waters than previously believed and should not be described as a purely coastal marine mammal. Our study also revealed notable interchange of individual seals between regions and countries. Conservation and management of the species needs to take this movement into account when considering population units. These new observations of at-sea distribution and connectivity may have an important bearing on our understanding of relationships among individuals primarily associated with different haul-out sites and how this affects susceptibility to epizootics and competition for prey, both of which are important for the conservation of a species in decline. The extensive distances travelled to forage mean that management of offshore fish stocks may be as important for harbour seals as management of inshore stocks.

That our results show that variation in foraging movements was better accounted for by region and season than by sex, size, and body condition, may also have an important bearing on harbour seal conservation. It suggests that management action to reverse population declines may be best focussed in an area-specific way. The EU Natura 2000 initiative to establish networks of Special Areas of Conservation (SACs) for habitats and species may be particularly relevant here (e.g. [53]). SACs have been designated for harbour seals around Britain but only for haul-out and pupping sites on land. Our results add weight to the impetus to determine and select sites at sea the nature of which should take into account regional observations of movement.

Finally, this study demonstrates the value of a large dataset collected over a wide spatial range, without which our results would be of little more than local interest. Instead, the range of habitats and population sizes studied makes our results relevant to the ecology and conservation of harbour seal populations worldwide. The expansion of our view of harbour seal distribution in British and surrounding waters would be further developed by spatial modelling of seals’ distribution. This could identify important features of high-use areas for harbour seals, which could also aid conservation populations of this species and marine conservation planning in general.

## Supporting Information

Appendix S1 **Tagging details for individual seals.**
(DOCX)

Appendix S2 **Regional trip distance and duration by sex.**
(DOCX)
