# Supplementary material for: Spatial Variation in Foraging Behaviour of a Marine Top Predator (Phoca vitulina) Determined by a Large-Scale Satellite Tagging Program
Source: PLoS One. 2012 May 21;7(5):e37216. doi: 10.1371/journal.pone.0037216 (PMC3357409; doi:10.1371/journal.pone.0037216)
Supplement: Appendix S1 — Tagging details for individual seals. (DOCX) [file pone.0037216.s001.docx]

**APPENDIX S1: Tagging details for individual seals**

| **Seal ID** | **Number of Locations** | **Capture Date** | **End Date** | **Tagging Duration (days)** | **Mass** | **Girth** | **Length** | **Sex** | |
| --- | --- | --- | --- | --- | --- | --- | --- | --- | --- |
| Outer Hebrides | | | | | | | | | |
| Pv18_Holly_06 | 928 | 14/03/2006 | 28/06/2006 | 106 | 96 | 111 | 152 | F | |
| Pv18_Izzy_06 | 1006 | 14/03/2006 | 09/06/2006 | 87 | 47 | 90 | 121 | F | |
| Pv18_Jemma_06 | 1311 | 14/03/2006 | 05/07/2006 | 113 | 82 | 106 | 137 | F | |
| Pv18_Mable_06 | 1378 | 14/03/2006 | 13/07/2006 | 121 | 78 | 103 | 136 | F | |
| Pv18_Kian_06 | 1270 | 15/03/2006 | 04/07/2006 | 111 | 84 | 110 | 152 | M | |
| Pv18_Edner_06 | 1022 | 16/03/2006 | 11/06/2006 | 81 | 86 | 110 | 145 | F | |
| Pv18_Freya_06 | 1307 | 16/03/2006 | 05/07/2006 | 123 | 86 | 105 | 142 | F | |
| Pv18_Laila_06 | 1162 | 16/03/2006 | 26/06/2006 | 108 | 94 | 115 | 148 | F | |
| Pv18_Don_06 | 914 | 17/03/2006 | 06/06/2006 | 179 | 113 | 129 | 154 | M | |
| Pv18_Garry_06 | 1234 | 17/03/2006 | 03/07/2006 | 122 | 110 | 123 | 150 | M | |
| Pv19a-Nancy-06 | 1359 | 18/09/2006 | 19/01/2007 | 123 | 76 | 103 | 144 | F | |
| Pv19g-Opal-06 | 1456 | 19/09/2006 | 17/03/2007 | 179 | 76 | 103 | 135 | F | |
| Pv19a-Quin-06 | 1381 | 20/09/2006 | 20/01/2007 | 122 | 79 | 98.5 | 147 | F | |
| Pv19g-Phil-06 | 1330 | 20/09/2006 | 18/02/2007 | 151 | 86 | 109 | 149 | M | |
| Pv19a-Saeed-06 | 912 | 23/09/2006 | 14/12/2006 | 82 | 79 | 105 | 152 | M | |
| Pv19g-Ross-06 | 623 | 23/09/2006 | 11/12/2006 | 79 | 98 | 108 | 152 | M | |
| Pv19a-Freya-06 | 1155 | 24/09/2006 | 03/01/2007 | 101 | 70 | 100 | 143 | F | |
| Pv19a-Tad-06 | 67 | 24/09/2006 | 03/10/2006 | 9 | 87 | 105 | 158 | M | |
| Pv19a-Ula-06 | 1683 | 25/09/2006 | 17/03/2007 | 173 | 73 | 106 | 144 | F | |
| Pv19a-Vern-06 | 1491 | 25/09/2006 | 19/02/2007 | 147 | 93 | 112 | 151 | M | |
| Shetland | | | | | | | | | |
| Pv1_Dink_03 | 1760 | 05/10/2003 | 10/03/2004 | 157 | 42.5 | 87 | 114 | M | |
| Pv1_foxy_03 | 2599 | 06/10/2003 | 06/06/2004 | 244 | 65 | 103 | 147 | F | |
| Pv1_gail_03 | 2801 | 06/10/2003 | 07/06/2004 | 245 | 79 | 110 | 142 | F | |
| Pv1_hatty_03 | 2254 | 06/10/2003 | 20/04/2004 | 197 | 84 | 106 | 146 | F | |
| Pv1_Clive_03 | 1339 | 07/10/2003 | 08/02/2004 | 124 | 90 | 113 | 155 | M | |
| Pv1_Erik_03 | 2626 | 07/10/2003 | 30/05/2004 | 236 | 91 | 112 | 140 | M | |
| Pv1_Frodo_03 | 173 | 07/10/2003 | 24/11/2003 | 48 | 102 | 113 | 150 | M | |
| Pv1_Gus_03 | 1133 | 07/10/2003 | 18/01/2004 | 103 | 117 | 122 | 160 | M | |
| Pv6_tina_04 | 1176 | 19/03/2004 | 11/07/2004 | 115 | 75 | 107 | 135 | F | |
| Pv6_ulli_04 | 1008 | 19/03/2004 | 29/06/2004 | 102 | 61 | 95 | 141 | F | |
| Pv6_val_04 | 884 | 19/03/2004 | 05/07/2004 | 108 | 108 | 120 | 148 | F | |
| Pv6_Quentin_04 | 1413 | 20/03/2004 | 20/08/2004 | 153 | 84 | 106 | 153 | M | |
| Pv6_wendy_04 | 1086 | 20/03/2004 | 08/07/2004 | 110 | 86 | 108 | 144 | F | |
| Pv6_Ned_04 | 269 | 21/03/2004 | 02/06/2004 | 73 | 100 | 118 | 151 | M | |
| Pv6_rona_04 | 1102 | 21/03/2004 | 30/06/2004 | 101 | 73 | 103 | 141 | F | |
| Orkney | | | | | | | | | |
| Pv1_ali_03 | 2045 | 01/10/2003 | 08/04/2004 | 190 | 87 | 109 | 138 | F | |
| Pv1_bo_03 | 2073 | 01/10/2003 | 04/04/2004 | 186 | 83.5 | 104 | 147 | F | |
| Pv1_cat_03 | 2191 | 01/10/2003 | 06/05/2004 | 218 | 66 | 108 | 148 | F | |
| Pv1_dot_03 | 2643 | 01/10/2003 | 11/07/2004 | 284 | 85 | 105 | 140 | F | |
| Pv1_Arnie_03 | 1631 | 02/10/2003 | 31/03/2004 | 181 | 84 | 110 | 146 | M | |
| Pv1_Bob_03 | 1413 | 02/10/2003 | 14/03/2004 | 164 | 88.5 | 108 | 152 | M | |
| Pv1_erin_03 | 1776 | 02/10/2003 | 16/03/2004 | 166 | 82.5 | 107 | 142 | F | |
| Pv6_Ken_04 | 1678 | 15/03/2004 | 05/08/2004 | 143 | 86 | 107 | 144 | M | |
| Pv6_Len_04 | 1206 | 15/03/2004 | 06/07/2004 | 113 | 103 | 114 | 154 | M | |
| Pv6_pat_04 | 1324 | 15/03/2004 | 01/09/2004 | 170 | 83 | 110 | 144 | F | |
| Pv6_Max_04 | 900 | 16/03/2004 | 02/06/2004 | 78 | 91 | 114 | 150 | M | |
| Pv6_queenie_04 | 1114 | 16/03/2004 | 23/06/2004 | 99 | 93.5 | 109 | 147 | F | |
| pv6_Oli_04 | 1052 | 18/03/2004 | 05/07/2004 | 109 | 87 | 103 | 153 | M | |
| pv6_Pete_04 | 249 | 18/03/2004 | 14/04/2004 | 27 | 121 | 124 | 164 | M | |
| pv6_sally_04 | 721 | 18/03/2004 | 30/05/2004 | 73 | 78 | 99 | 146 | F | |
| Moray Firth | | | | | | | | | |
| pv9_Angus_04 | 424 | 29/09/2004 | 09/12/2004 | 71 | 78 | 98 | 144 | M | |
| pv9_Burt_04 | 361 | 29/09/2004 | 16/11/2004 | 48 | 78 | 103 | 146 | M | |
| pv9_Chris_04 | 1005 | 29/09/2004 | 02/04/2005 | 185 | 77 | 102 | 148 | M | |
| pv9_dory_04 | 771 | 16/10/2004 | 20/03/2005 | 155 | 60 | 97 | 138 | F | |
| pv9_Gabe_04 | 746 | 16/10/2004 | 14/03/2005 | 149 | 68 | 107 | 145 | M | |
| pv11_Helen_05 | 842 | 05/03/2005 | 25/05/2005 | 81 | 71 | 100 | 136 | F | |
| pv11_Isla_05 | 1191 | 05/03/2005 | 28/07/2005 | 145 | 79 | 108 | 133 | F | |
| pv11_James_05 | 320 | 05/03/2005 | 06/04/2005 | 32 | 70 | 103 | 142 | M | |
| pv11_Kath_05 | 1077 | 06/03/2005 | 22/07/2005 | 138 | 80 | 112 | 135 | F | |
| pv11_Lewis_05 | 1212 | 06/03/2005 | 22/07/2005 | 138 | 87.5 | 115 | 148 | M | |
| St Andrews | | | | | | | | | |
| ab2_Albert_01 | 1690 | 02/11/2001 | 17/04/2002 | 166 | 68 | 90 | 138 | M | |
| ab2_Barry_01 | 962 | 02/11/2001 | 27/01/2002 | 86 | 69 | 87 | 144 | M | |
| ab2_Chester_01 | 1294 | 19/11/2001 | 23/03/2002 | 124 | 76 | 112 | 142 | M | |
| ab2_Denzil_01 | 1446 | 20/11/2001 | 26/04/2002 | 157 | 59 | 94 | 125 | M | |
| ab2_Ellie_01 | 1580 | 16/01/2002 | 26/04/2002 | 100 | 66 | 102 | 137 | F | |
| ab2_Fred_01 | 1328 | 16/01/2002 | 21/06/2002 | 156 | 58.5 | 99 | 125 | M | |
| ab2_Georgia_01 | 1036 | 16/01/2002 | 19/05/2002 | 123 | 82 | 106 | 144 | F | |
| ab2_Harriot_01 | 1049 | 16/01/2002 | 02/05/2002 | 106 | 76 | 108 | 145 | F | |
| ab2_Iona_01 | 1228 | 16/01/2002 | 01/05/2002 | 105 | 75 | 108 | 137 | F | |
| ab2_Jen_01 | 1789 | 16/01/2002 | 26/05/2002 | 130 | 72 | 101 | 142 | F | |
| ab2_Kelly_02 | 1378 | 24/10/2002 | 06/05/2003 | 194 | 50 | 85 | 121 | F | |
| ab2_Leroy_02 | 1228 | 24/10/2002 | 26/04/2003 | 184 | 64 | 97 | 136 | M | |
| ab2_Marty_02 | 1298 | 24/10/2002 | 20/02/2003 | 119 | 88 | 109.5 | 143 | M | |
| ab2_Noris_02 | 1165 | 24/10/2002 | 07/03/2003 | 134 | 71 | 102 | 141 | M | |
| ab2_Otis_02 | 1185 | 24/10/2002 | 23/03/2003 | 150 | 78 | 101 | 143 | M | |
| ab2_Pedro_02 | 1625 | 24/01/2003 | 01/07/2003 | 158 | 77 | 96 | 132 | M | |
| ab2_Quincy_02 | 1313 | 24/01/2003 | 04/06/2003 | 131 | 87 | 104 | 126 | M | |
| ab2_Ronda_02 | 1356 | 11/03/2003 | 22/07/2003 | 133 | 51.5 | 90 | 123 | F | |
| ab2_Sooz_03 | 1452 | 11/03/2003 | 22/07/2003 | 133 | 67 | 96 | 135 | F | |
| ab2_Tigger_03 | 977 | 11/03/2003 | 08/06/2003 | 89 | 52 | 91 | 120 | M | |
| ab2_Ursula_03 | 1121 | 11/03/2003 | 18/07/2003 | 129 | 75 | 109 | 127 | F | |
| ab2_Vince_02 | 1037 | 11/03/2003 | 14/06/2003 | 95 | 82 | 106 | 138 | M | |
| ab2_Wilma_03 | 1367 | 25/03/2003 | 27/07/2003 | 124 | 92 | 111 | 134 | F | |
| ab2_Xena_03 | 1139 | 25/03/2003 | 07/07/2003 | 104 | 72 | 110 | 131 | F | |
| ab2_Yoko_02 | 1001 | 25/03/2003 | 21/06/2003 | 88 | 63.5 | 96 | 139 | F | |
| The Wash | | | | | | | | |  |
| pv3_Harry_03 | 1209 | 24/10/2003 | 28/03/2004 | 156 | 51 | 94 | 128 | M | |
| pv3_Isaac_03 | 718 | 24/10/2003 | 08/01/2004 | 76 | 68 | 97 | 143 | M | |
| pv3_isabel_03 | 1219 | 24/10/2003 | 01/06/2004 | 221 | 73 | 108 | 140 | F | |
| pv3_jade_03 | 1438 | 24/10/2003 | 31/03/2004 | 159 | 68 | 104 | 142 | F | |
| pv3_lilly_03 | 1480 | 25/10/2003 | 12/05/2004 | 200 | 71 | 104 | 142 | F | |
| pv4_Jed_04 | 1274 | 18/02/2004 | 23/07/2004 | 156 | 89 | 111 | 141 | M | |
| pv4_Kay_04 | 1143 | 18/02/2004 | 31/07/2004 | 164 | 100 | 119 | 142 | F | |
| pv4_mary_04 | 1164 | 18/02/2004 | 15/08/2004 | 179 | 100 | 120 | 140 | F | |
| pv4_nina_04 | 1420 | 18/02/2004 | 03/07/2004 | 136 | 93.5 | 117 | 139 | F | |
| pv4_ode_04 | 878 | 18/02/2004 | 13/06/2004 | 116 | 93.5 | 112 | 140 | F | |
| pv9_apple_04 | 521 | 10/10/2004 | 07/02/2005 | 120 | 73 | 103 | 131 | F | |
| pv9_bell_04 | 655 | 10/10/2004 | 05/03/2005 | 146 | 72 | 106 | 139 | F | |
| pv9_clare_04 | 578 | 10/10/2004 | 08/02/2005 | 121 | 65.5 | 101 | 134 | F | |
| pv9_Dom_04 | 944 | 10/10/2004 | 30/03/2005 | 171 | 73 | 101 | 138 | M | |
| pv9_Edd_04 | 539 | 10/10/2004 | 01/02/2005 | 114 | 89.5 | 114 | 142 | M | |
| pv9_Fluff_04 | 495 | 10/10/2004 | 08/04/2005 | 180 | 67 | 100 | 133 | M | |
| pv15_Moss_05 | 906 | 21/03/2005 | 24/06/2005 | 95 | 89 | 115 | 145 | M | |
| pv15_Nevil_05 | 1067 | 21/03/2005 | 21/07/2005 | 122 | 88.5 | 118 | 146 | M | |
| pv15_Owen_05 | 1049 | 21/03/2005 | 15/10/2005 | 208 | 101 | 117 | 142 | M | |
| pv15_Poppy_05 | 668 | 21/03/2005 | 06/06/2005 | 77 | 74.5 | 108 | 145 | F | |
| pv15_Q_05 | 757 | 21/03/2005 | 23/07/2005 | 124 | 93.5 | 116 | 157 | M | |
| pv15_Romeo_05 | 439 | 21/03/2005 | 03/07/2005 | 104 | 90 | 113 | 145 | M | |
| pv15_Sonja_05 | 1249 | 22/03/2005 | 18/08/2005 | 149 | 95 | 116 | 141 | F | |
| pv15_Tracy_05 | 597 | 22/03/2005 | 28/05/2005 | 67 | 64 | 99 | 130 | F | |
| The Thames | | | | | | | | | |
| pv17-Aiden-06 | 1728 | 19/02/2006 | 31/07/2006 | 162 | 90 | 116 | 141 | M | |
| pv17-Brett-06 | 1116 | 19/02/2006 | 17/06/2006 | 118 | 95 | 119 | 143 | M | |
| pv17-Callan-06 | 1384 | 21/02/2006 | 10/07/2006 | 139 | 94 | 111 | 150 | M | |
| pv20a-Barny-06 | 1348 | 12/10/2006 | 22/02/2007 | 133 | 78 | 108 | 148 | M | |
| pv20a-Waldo-06 | 1537 | 12/10/2006 | 09/03/2007 | 148 | 68 | 103 | 148 | M | |
| pv20a-Xenon-06 | 1353 | 12/10/2006 | 20/02/2007 | 131 | 68 | 101.5 | 147 | M | |
| pv20a-Yogi-06 | 1096 | 12/10/2006 | 09/02/2007 | 120 | 75 | 106.5 | 149 | M | |
| pv20a-Zack-06 | 673 | 12/10/2006 | 25/12/2006 | 74 | 73 | 107 | 136 | M | |
| pv20g-Adam-06 | 730 | 12/10/2006 | 28/12/2006 | 77 | 66 | 101 | 144 | M | |
