# Supplementary material for: Spatial Variation in Foraging Behaviour of a Marine Top Predator (Phoca vitulina) Determined by a Large-Scale Satellite Tagging Program
Source: PLoS One. 2012 May 21;7(5):e37216. doi: 10.1371/journal.pone.0037216 (PMC3357409; doi:10.1371/journal.pone.0037216)
Supplement: Appendix S2 — Regional trip distance and duration by sex. (DOCX) [file pone.0037216.s002.docx]

**APPENDIX S2: Regional trip distance and duration by sex**

| **Region** | **N(trips)** | |  | **Trip distance (km)** | |  |  | **Trip duration (days)** | |  |
| --- | --- | --- | --- | --- | --- | --- | --- | --- | --- | --- |
|  | **Male** | **Female** | **Male** | **SD** | **Female** | **SD** | **Male** | **SD** | **Female** | **SD** |
| Moray Firth | 65 | 77 | 152.64 | 166.92 | 56.67 | 59.19 | 6.78 | 6.87 | 3.60 | 3.62 |
| Orkney | 279 | 598 | 18.98 | 26.34 | 21.97 | 65.14 | 1.53 | 1.60 | 1.45 | 1.73 |
| Outer Hebrides | 286 | 591 | 35.02 | 61.34 | 25.16 | 28.78 | 1.59 | 2.60 | 1.45 | 1.34 |
| Shetland | 361 | 479 | 10.79 | 29.54 | 11.31 | 32.74 | 1.31 | 1.51 | 1.50 | 1.74 |
| St Andrews | 270 | 289 | 76.73 | 104.42 | 36.94 | 51.90 | 3.99 | 4.25 | 2.62 | 3.38 |
| Thames | 649 | 0 | 17.00 | 28.62 | - | - | 0.90 | 0.97 | - | - |
| The Wash | 220 | 220 | 77.30 | 112.33 | 94.97 | 110.38 | 3.73 | 5.33 | 4.50 | 5.14 |
